# Genetic Diversity in New Members of the Reticulocyte Binding Protein Family in Thai *Plasmodium vivax* Isolates

**DOI:** 10.1371/journal.pone.0032105

**Published:** 2012-03-05

**Authors:** Varakorn Kosaisavee, Usa Lek-Uthai, Rossarin Suwanarusk, Anne Charlotte Grüner, Bruce Russell, Francois Nosten, Laurent Rénia, Georges Snounou

**Affiliations:** 1 Department of Parasitology and Entomology, Faculty of Public Health, Mahidol University, Bangkok, Thailand; 2 Doctor of Public Health Programme (Parasitology), Faculty of Graduate Studies, Mahidol University, Nakornpathom, Thailand; 3 Singapore Immunology Network, Agency for Science, Technology and Research, Biopolis, Singapore; 4 Shoklo Malaria Research Unit, Mae Sot, Tak, Thailand; 5 Center for Clinical Vaccinology and Tropical Medicine, Oxford, United Kingdom; 6 Institut National de la Santé et de la Recherche Médicale, Unité Mixte de Recherche S 665, Paris, France; 7 Université Pierre & Marie Curie, Faculté de Médecine Pitié-Salpêtrière, Paris, France; London School of Hygiene and Tropical Medicine, United Kingdom

## Abstract

**Background:**

*Plasmodium vivax* merozoites specifically invade reticulocytes. Until recently, two reticulocyte-binding proteins (*Pvrbp1* and *Pvrbp2*) expressed at the apical pole of the *P. vivax* merozoite were considered to be involved in reticulocyte recognition. The genome sequence recently obtained for the Salvador I (Sal-I) strain of *P. vivax* revealed additional genes in this family, and in particular *Pvrbp2a*, *Pvrbp2b* (*Pvrbp2* has been renamed as *Pvrbp2c*) and two pseudogenes *Pvrbp2d* and *Pvrbp3*. It had been previously found that *Pvrbp2c* is substantially more polymorphic than *Pvrbp1*. The primary goal of this study was to ascertain the level of polymorphism of these new genes.

**Methodology/Principal Findings:**

The sequence of the *Pvrbp2a*, *Pvrbp2b*, *Pvrbp2d* and *Pvrbp3* genes were obtained by amplification/cloning using DNA purified from four isolates collected from patients that acquired the infection in the four cardinal regions of Thailand (west, north, south and east). An additional seven isolates from western Thailand were analyzed for gene copy number variation. There were significant polymorphisms exhibited by these genes (compared to the reference Sal-I strain) with the ratio of mutations leading to a non-synonymous or synonymous amino acid change close to 3∶1 for *Pvrbp2a* and *Pvrbp2b*. Although the degree of polymorphism exhibited by these two genes was higher than that of *Pvrbp1*, it did not reach the exceptional diversity noted for *Pvrbp2c*. It was interesting to note that variations in the copy number of *Pvrbp2a* and *Pvrbp2b* occurred in some isolates.

**Conclusions/Significance:**

The evolution of different members of the *Pvrbp2* family and their relatively high degree of polymorphism suggests that the proteins encoded by these genes are important for parasite survival and are under immune selection. Our data also shows that there are highly conserved regions in *rbp2a* and *rbp2b*, which might provide suitable targets for future vaccine development against the blood stage of *P. vivax*.

## Introduction

*Plasmodium* spp. merozoites invade the host red cell via a multistep invasion process [1]. Parasite species differ in the preference they exhibit with respect to the type of red blood cells they can invade, for example *P. malariae* is almost exclusively observed in normocytes, while *P. vivax* is confined to reticulocytes. Thus, one of the initial steps prior to invasion is the specific recognition of the red blood cell type. For *P. vivax* two proteins expressed at the apical pole of the merozoite have been identified and implicated in reticulocyte recognition/selection, and were named reticulocyte binding proteins (RBP) [2]. Genes related to those coding for the RBPs of *P. vivax* (*Pvrbp*) were found in *Plasmodium* species that infect humans [3], [4], [5], [6], simians [7], [8], [9] and rodents [10], [11], [12]. Given their crucial implication in the invasion process, PVRBP proteins are considered to be good vaccine candidates [1], [13], [14]. However, investigations aiming to define the functional domains of *Pvrbp* genes or their receptors on the reticulocyte were hampered by the fact that a practical invasion assay for *P. vivax* invasion have been developed only recently [15], and that the two genes are quite large in size (ca. 8 kb–9 kb). Thus, overlapping peptides were used to define the binding domains of *Pvrbp1* [16], [17], [18]. The other investigation was confined to an assessment of *Pvrbp1* and *Pvrbp2* diversity in four isolates from different geographical regions [19], which revealed a remarkably high diversity in *Pvrbp2* as compared to *Pvrbp1* and its homolog in *P. falciparum* and provided an indication as to the sub-domains it might be worth to focus on in future studies.

When the complete genome of the Salvador I strain of *P. vivax* was obtained [20], it was noted that there were other *Pvrbp* genes present, which led to the reclassification of the *Pvrbp* family (Table 1). There were two partial genes, and 7 full-length genes of which two were pseudogenes (*Pvrbp2d* and *Pvrbp3*). The primary aim of this study was to ascertain whether the high sequence diversity observed for *Pvrbp2c* (the new name for the gene previously known as *Pvrbp2*) also characterises the newly uncovered members of the *Pvrbp* family.

**Table 1:** Chromosomal location of the *Plasmodium vivax* Reticulocyte Binding Protein genes.

Name	Strain	Chromosome
*Pvrbp1a*	Sal-I	7
*Pvrbp1b*	Sal-I	7
*Pvrbp2a*	Sal-I	14
*Pvrbp2b*	Sal-I	8
*Pvrbp2c*	Sal-I	5
*Pvrbp2* (partial)	Sal-I	5
*Pvrbp2d*	Sal-I	14
*Pvrbp2* (partial)	Sal-I	14
*Pvrbp3*	Sal-I	14

## Materials and Methods

### Ethics

This study was approved from Human Research Ethical Committee at Ministry of Public Health, Thailand (Reference no. 101/2550). This study utilized *P. vivax* infected blood samples collected prior to treatment and after informed and written consent from patients presenting in 2008–2009 at clinics from Tak, Mae Hong Sorn, Prachup Kririkhan or Chantaburi provinces, which are located in west, north, south and east Thailand, respectively.

### Blood samples

Approximatley30 µl of blood were spotted onto filter paper (Protein saver, Whatman®) and kept in a dry and cool place until DNA extraction. Giemsa stained blood films were used to determine the species present and the parasitaemia. DNA from dried blood spots was extracted using QIAamp DNA Blood Mini Kit (QIAGEN, Germany) following the manufacturer's instructions, and the resulting DNA templates were stored at −20°C. Four samples, one from each region were selected for use in this study, after they were confirmed by PCR [21] to contain only *P. vivax*, and where parasitaemias were close to 400 parasites per microlitre of blood (about 1 parasites per 1000 RBC). Seven additional samples (numbered TK1 to TK7) were randomly selected from the west of Thailand (Tak province) for determination of the *pvrbp2a* and *pvrbp2b* copy number.

### Reticulocyte Binding Protein (RBP) primer design and amplification

Reticulocyte Binding Protein (*rbp* genes) primers were designed based on the sequences obtained from the *P. vivax* Sal-I genome: Gene ID PVX_121920, PVX_094255, PVX_101585, and PVX_101495, which code for *Pvrbp2a*, *Pvrbp2b*, *Pvrbp2d* (pseudogene) and *Pvrbp3* (pseudogene), respectively [22]. Given their large size (around eight kb), six overlapping 1.5 kb PCR fragments were amplified for each gene (eight fragments for the 8.7 kb *Pvrbp*3 gene). All primers and annealing temperature are shown in (Table S1). Two microlitres of the DNA template were used to initiate PCR amplification that was carried out in a total volume of 20 µl in the presence of 30 mM Tris-HCl, pH 8.3, 100 mM KCl, 2 mM MgCl_2_, 0.25 µM primers, 250 µM dNTPs and 0.02 U/µl of Ampli Taq Gold DNA Polymerase (Applied Biosystems). The cycling conditions were an initial denaturation 94°C for 5 minutes; followed by 35 cycles: annealing at a defined temperature (see Table S1) for 2 minutes, extension at 72°C for 2 minutes, denaturation 94°C for 1 minute; and a final extension at 72°C for 20 minutes. PCR products were analysed on an agarose gel stained with Gel red (Biotium, Inc., CA, USA) and visualized under UV light. All amplicons were immediately stored in −20°C until cloning process.

### RBP cloning and sequence analysis

The PCR products from each gene were cloned into pCR4 Topo vector (Topo® TA cloning Kit, Invitrogen), and the resulting plasmids were sequenced using BigDye v3.1 Terminator (Applied Biosystems) and analyzed on an ABI 730XL (Applied Biosystems). Sequences were generated from both forward and reverse strands. Only single nucleotide polymorphisms (SNPs) that were observed in two or more fragments obtained from independent PCR amplifications were considered to be confirmed and unlikely to result as an artefact of the fidelity of the polymerase.

### Copy number determination

The copy number of *Pvrbp2a* and of *Pvrbp2b* gene was estimated by a quantitative real-time SYBR Green PCR assay, as previously described [23]. Primers for these two genes were designed according to their conserved regions (Table S2). The qPCR method utilized a plasmid [vector pCR2.1® (Topo TA cloning kit, Invitrogen)] containing the reference *P. vivax* aldolase gene (GenBank Acc. No. AF247063) (single copy standard), and the gene of interest. Real-time PCR was run in the LightCycler® 480 (Roche Applied Science) using the following thermocycler program: 10 minutes of pre-incubation at 95°C before conducting 45 cycles of 10 seconds at 95°C, 5 seconds at 60°C and 10 seconds at 60°C. Subsequent to this, the melting curve was set for 0 second initiating temperature at 95°C, 15 seconds at 60°C, continuous at 72°C and cool down at 40°C 30 seconds. The reaction volume for each sample was 20 µl, and the reactions were placed in a 96-well plate (Applied Biosystems™) in the presence of 1× SYBRGreen buffer (Roche Applied Science), 2.5 mM MgCl_2_, 250 nM of each primer, and 2 µl of DNA. At the end of each reaction, Cycle threshold (Ct) was evaluated and melting curves were acquired and analyzed. The ΔΔCt calculation for the relative quantification of *P. vivax* gene target was used as follows ΔΔCt = Ct selected gene−Ct aldolase. The gene copy number in each isolate was calculated in N-fold changes by using equation as showing: N-fold copies in isolate = 2^−ΔΔCt^ [24].

## Results and Discussion

**Figure 1:**
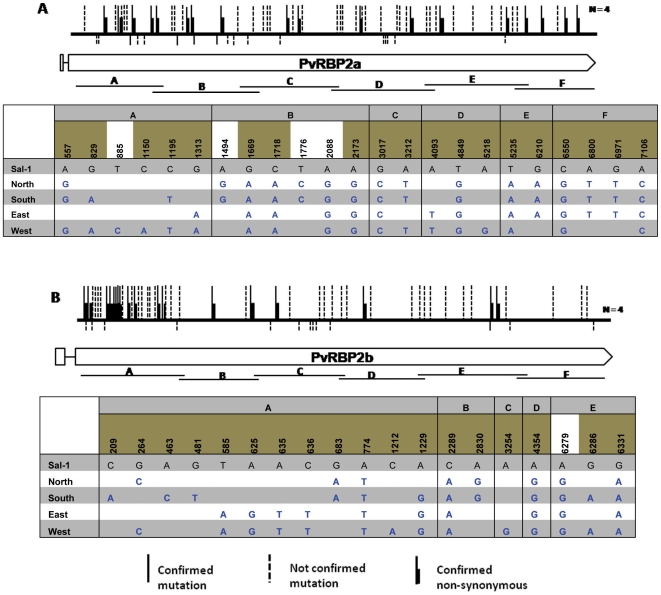
Schematic representation of the *Pvrbp2a* (A) and *Pvrbp2b* (B) genes. In each panel, the location of the fragments cloned is indicated below the gene model. Above the gene model, synonymous mutations are indicated by vertical bars below the horizontal bar that represents the gene, whereas non-synonymous mutations are place above this horizontal bar. SNPs that have been confirmed from two independent PCR amplifications are shown as solid lines, whereas those observed only once are represented by dotted lines. The nature and location of confirmed SNPs are also provided as tables, with the non-synonymous mutations highlighted in olive green.

**Figure 2:**
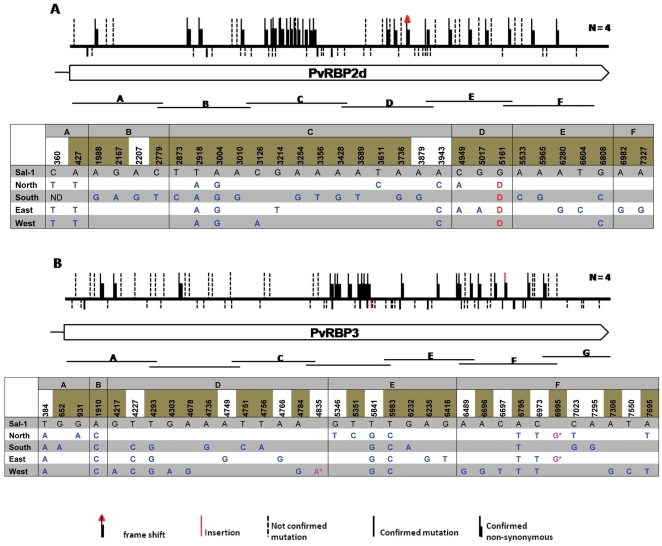
Schematic representation of the *Pvrbp2d* (A) and *Pvrbp3* (B) genes. In each panel, the location of the fragments cloned is indicated below the gene model. Above the gene model, synonymous mutations are indicated by vertical bars below the horizontal bar that represents the gene, whereas non-synonymous mutations are place above this horizontal bar. SNPs that have been confirmed from two independent PCR amplifications are shown as solid lines, whereas those observed only once are represented by dotted lines. The nature and location of the confirmed SNPs are also provided in the tables, with the non-synonymous mutations highlighted in olive green. As these two genes are pseudogenes, the consequence of the SNP, synonymous or non-synonymous, has been predicted assuming that a continuous reading frame.

The sequences of the four recently discovered members of the *Pvrbp* genes (*Pvrbp2a*, *Pvrbp2b*, *Pvrbp2d*, and *Pvrbp3*) (Table 1) were obtained from 4 *P. vivax* isolates collected from distinct regions of Thailand (Accession numbers JN122379–JN122425 & JN172857–JN172908; totalling 130.5 kb of sequences). In all four *P. vivax* isolates from Thailand the *Pvrbp2d*, *Pvrbp3* were confirmed to be pseudogenes, as was found for those in Sal-I. The sequences obtained for all the genes were then aligned with those of the *P. vivax* Sal-I strain genes. The overall results of this analysis are presented in Table 2, and schematically in Fig. 1 & 2. The sequences of the genes from the Thai *P. vivax* isolates differed significantly from those of the Sal-I strain with about 8 to 13 SNPs per 1000 bp. This level of diversity is closer to that observed for *Pvrbp1* (3.3 SNP per 1000 bp) than to that for *Pvrbp2c* (76.4 SNPs per 1000 bp) (Table 2). It should however, be pointed out that more than half of the SNP's detected were observed only once, and it is possible that some were artefacts of the amplification reaction. Confirmation by sequencing of all the gene fragments from two independent PCR amplifications could not be carried out for logistic regions: this would have represented an extra 130 kb of sequences to obtain). If only the confirmed SNP'a are taken into consideration, then the level of diversity (3 to 4 SNPs per 1000 bp) nears that found previously for *Pvrbp1* [19] (Table 2). The ratio of non-synonymous (NS) to synonymous (S) mutations for *Pvrbp2a* and *Pvrbp2b* was 3.0 and 3.1, respectively, which was comparable to that noted for *Pvrbp2c* (2.4 and 3.9) and lower than that recorded (4.5) for *Pvrbp1* [19] (Table 2). The NS/S ratios for the two pseudogenes *Pvrbp2d* and *Pvrbp3*, was 1.2 and 1.4, respectively, as would be expected for non-functional genes. The extent of diversity observed was more restricted between the genes of the four isolates from Thailand, as compared to that noted when the Sal-I sequences were considered. It should also be noted that for all the mutations observed for any particular residue, the nucleotide substituted was invariably the same in the genes from the four isolates (except for one synonymous SNP at position 7023 of the *Pvrbp3* pseudogene). There was some degree of clustering of the non-synonymous mutations in *Pvrbp2b*, suggesting that different domains might be under differential selective pressure. However, a similar pattern of clustering was observed for the two pseudogenes.

**Table 2:** Genetic diversity within the *Plasmodium vivax* Reticulocyte binding protein genes.

Gene	n	Size	SNP (n)		Reference
			NS	S	
***Pvrbp1***	4	8499	25	3	Rayner *et al.* [19]
***Pvrbp*** **2c**	4	8454	489	151	Rayner *et al.* [19]
***Pvrbp2a***	4	7464	55	18	This study
***Pvrbp2b***	4	7959	51	13	This study
***Pvrbp2d***	4	8495	34	28	This study
***Pvrbp3***	4	8702	67	48	This study

Note: n = number of sequenced isolates; Size = size of the gene analyzed with the non-coding regions excluded; SNP (n) = number of single nucleotide polymorphisms; NS = non-synonymous substitutions; S = synonymous substitutions.

**Figure 3:**
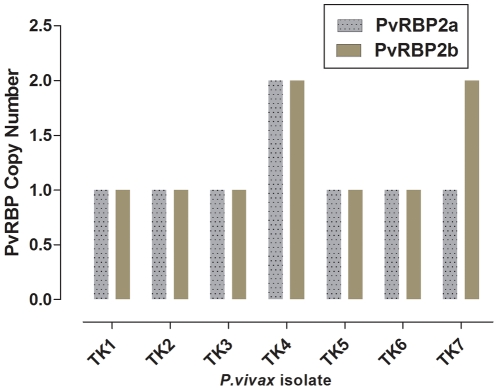
The copy number of *Pvrbp2a*, *Pvrbp2b* genes in seven *P. vivax* isolates (Numbered TK1 to TK7) collected in western Thailand (Tak province).

It had been previously noted that some *P. falciparum* lines harbour multiple copies of one of the *Pvrbp* homologues, *Pfrh1* [25]. Variation in the copy number of *Pvrbp2a* and *Pvrbp2b* were sought in seven isolates collected from west Thailand (Tak province), and this was found in two of these *P. vivax* isolates: there were two copies of *Pvrbp2b* in one, and there were two copies of both *Pvrbp2a* and *Pvrbp2b* in the other (Fig. 3). The functional significance, if any, of these copy number variations is not known at present. This would also require a survey of a larger number of *P. vivax* isolates, and ultimately a means to ascertain if there is a link between the number of *Pvrbp* copy number and a particular parasite phenotype. Nonetheless, one could speculate that the pseudogenes (*Pvrbp2d* and *Pvrbp3*) might have been derived from genes present as multiple copies. It should be confirmed that *Pvrbp2d* and *Pvrbp3* are indeed true pseudogenes. For example, evidence for the protein encoded by one of the homologues of the *Pvrbp* genes in *P. falciparum* (*Pfrh3*) that was considered to be a pseudogene, was detected by proteomic analysis in sporozoites extracts [26].

Given that the sequence data presented was derived from four isolates only, it is felt that any conclusions that can be drawn from the pattern of mutations are at best speculative. For example, it would be interesting to investigate whether a higher degree of genetic diversity would be observed for *P. vivax* from the west of Thailand as a result of the relatively higher incidence of this parasites as compared to the other areas of Thailand (Malaria Cases Report (Report 506): Annual report for the fiscal year 2006–2007 by provinces, Department of Communicable Disease Control, Ministry of Public Health, Bangkok 2008). Similarly, it is not known whether the nature and extent of the diversity observed is specific to the local parasite populations: *P. vivax* circulating in Thailand as compared to those circulating in geographically isolated populations of *P. vivax*, such as; Papua New Guinea, Afghanistan, Madagascar or South America. Until such a time when cloning and sequencing of high numbers of large genes becomes economically justifiable, one can envisage studies that target particular sub-domains of the *Pvrbp* genes.

### Conclusion and Future Studies

To our knowledge, this is the first investigation of the genetic diversity of the recently uncovered full-length members of the *Pvrbp* family [20]. There is still very little known about the specific role of the corresponding proteins. Nonetheless, the high number of non-synonymous mutations in *rbp2a* and *rbp2b* suggest that they are likely to be under active selection and occurrence of *P. vivax* strains in which one or both are present as multiple copies is consistent with an important role in the survival of the parasite. A recently validated invasion inhibition assay employing *P. vivax* field isolates [15] makes it possible to envisage investigations of the functional role of the various PvRBP proteins or domains thereof, and ultimately to establish if any can serve as a useful target for a future vaccine against *P. vivax*.

## Supporting Information

Table S1 **Sequence and annealing temperature of the primers used to amplify the** ***Pvrbp*** **genes.**
(DOCX)

Table S2 **Primers set for** ***Pvrbp*** **genes copy number determination.**
(DOCX)
