# Correction: Genetic Diversity in New Members of the Reticulocyte Binding Protein Family in Thai *Plasmodium vivax* Isolates

**DOI:** 10.1371/annotation/4e852b7d-735e-436f-bc66-3c31706b5f26

**Published:** 2012-06-13

**Authors:** Varakorn Kosaisavee, Usa Lek-Uthai, Rossarin Suwanarusk, Anne Charlotte Grüner, Bruce Russell, Francois Nosten, Laurent Rénia, Georges Snounou

The published affiliation of Dr Laurent Rénia is incorrect. The correct affiliation of Laurent Rénia is:

3 Singapore Immunology Network, Agency for Science, Technology and Research, Biopolis, Singapore. 

