# Supplementary material for: Genetic Diversity in New Members of the Reticulocyte Binding Protein Family in Thai Plasmodium vivax Isolates
Source: PLoS One. 2012 Mar 5;7(3):e32105. doi: 10.1371/journal.pone.0032105 (PMC3293883; doi:10.1371/journal.pone.0032105)
Supplement: Table S1 — Sequence and annealing temperature of the primers used to amplify the Pvrbp genes. (DOCX) [file pone.0032105.s001.docx]

**Supplement Table 1.** Sequence and annealing temperature of the primers used to amplify the *Pvrbp* genes.

| **Name** | **Sequence 5’ to 3’** | **Annealing (^o^C)** | **Product (bp)** |
| --- | --- | --- | --- |
| ***rbp2a*_A** | F-GGAACCTAAGCTATGGCCACTACAAG  R- GAGTTTTCTTTGCTTGCTTCTGTTC | 58 | 1371 |
| ***rbp* 2*a*_B** | F-GAGCCGATACCACGACAGCTGCGC  R-CCAGTTTTGCGTTTATATTTTCATTGC | 54 | 1485 |
| ***rbp* 2*a*_C** | F-CTTGGCGCACATAGATACTACTAC  R-CTCCTCTGCTTTTGCACTTTTATC | 54 | 1384 |
| ***rbp* 2*a*_D** | F-GAAGAGATTAAAACAACTATAGCAAGC  R-GTCCAATCCATTAAAGAGGGAAGC | 58 | 1458 |
| ***rbp* 2*a*_E** | F-ATTTCCTCCGATACGGATAACATAG  R-CTAAATTTTGTAGGAGTTTTACTTGCTC | 58 | 1240 |
| ***rbp* 2*a*_F** | F-AATTGATAACGCTACTACGCTGCTGC  R-CATCACGTGAATTTAGATCTCTTCC | 58 | 1298 |
| ***rbp* 2*b*_A** | F-CGCATCATATCACACAGAAATCAAAC  R-CGCTCGTGAAATGTATGCATTATTTC | 60 | 1512 |
| ***rbp* 2*b*_B** | F-CCAACAACATGTGGAAGAATTAATCAAC  R-CCTGTATACGTTGCTCAACTTTAAGCTC | 60 | 1508 |
| ***rbp* 2*b*_C** | F-CGCAAGCGAAATTGAGTGGGAAGCTAC  R-CCCCCTCAATTTGGCCCATTATTTTGG | 60 | 1538 |
| ***rbp* 2*b*_D** | F-GCCGACTGTGTGGAACACTCAAAAG  R-GTAGGCGGCTTGCACATTTTCGTGGAC | 60 | 1422 |
| ***rbp* 2*b*_E** | F-GAGCCTAAACGATGAAAGTGGCTCG  R-CGCTTTAATGGACACTAGGGTGAAGG | 60 | 1477 |
| ***rbp* 2*b*_F** | F-GCTTGAAATAAGAGCCACCTCTG  R-CCTTCAAATTGTTCCCCGACGGATTC | 60 | 1413 |
| ***rbp* 2*d*_A** | F-CAGCATCATGTAAGGATGTCAATCG  R-CATGTTGTGCTATTTCTTTAGAACTCG | 61 | 1465 |
| ***rbp* 2*d*_B** | F-CATCCAAAAGTAAAGAACAGGTAC  R-CTCTGCTATTGTTGGCGCTGTTTCTAG | 61 | 1584 |
| ***rbp* 2*d*_C** | F-GGAGCGAACGCCGATGAACAACATCAAG  R-GCCTTAGTTATCAAATCTCTGATTGCTTG | 61 | 1538 |
| ***rbp* 2*d*_D** | F-CCAATCAGAAAATAGTTCTATAGAGG  R-GTTTAATGTATTCATATCCATCATTAACC | 61 | 1551 |
| ***rbp* 2*d*_E** | F-CCCTAGCGAATGAGCTGAGGAAAGAAGCG  R-CTACATCCAAATATTTATCAGCATCTG | 61 | 1551 |
| ***rbp* 2*d*_F** | F-GAAACGATAAGCGATCATGCGCCAAC  R-GCTTCTTCTTGTTCCTTTTCTATGGC | 61 | 1434 |
| ***rbp* 3_A** | F-CGGATGGTGGGGAGTATGCTTCGGGG  R-GCCAAGTCGAAACTTTTCTCCACCAAGC | 61 | 1450 |
| ***rbp* 3_B** | F-CTGGGACAAGCATTGTTAGCATTATAGA  R-CCCCCTTTAGTATAACCGTTTTGTATTC | 61 | 1446 |
| ***rbp* 3_C** | F-CAGAATCTTTGCAGAACATAACGAATG  R-CTGCCATTTCCTGCTCACACTTTTTCTG | 61 | 1506 |
| ***rbp* 3_D** | F-CAAGGCCAAGGAAAAGGCCATGAAAG  R-CACATCCATGTTGCTCTTCTCTTCCG | 61 | 1465 |
| ***rbp* 3_E** | F- GAATTAGTCGAGTCTAATTTAGGCCAG  R-CCGTCGACCTCCGTCTCGGATACTTC | 61 | 1412 |
| ***rbp* 3_F** | F-CGAAGGGTGGATAAGGAAAGGGAAC  R-CCCCCACTTGAAAGTAGTTATCATTTTC | 61 | 1517 |
| ***rbp* 3_G** | F-CGATGTAGTAAGCCATAGTGCGGCG  R-CTTCAATAATTTCCTCCTTTTCCCG | 61 | 1166 |

* Amplification performed at a final concentration of 3 mM MgCl_2_
