# Supplementary material for: Genetic Diversity in New Members of the Reticulocyte Binding Protein Family in Thai Plasmodium vivax Isolates
Source: PLoS One. 2012 Mar 5;7(3):e32105. doi: 10.1371/journal.pone.0032105 (PMC3293883; doi:10.1371/journal.pone.0032105)
Supplement: Table S2 — Primers set for Pvrbp genes copy number determination. (DOCX) [file pone.0032105.s002.docx]

**Supplement Table 2.** Primers set for *Pvrbp* genes copy number determination.

| **Name** | **sequence (5'----3')** |
| --- | --- |
| ***rbp2a* F** | GAAATAAGGGAGAAAACCGACG |
| ***rbp2a* R** | CTAAGATGATTTTAGACCTTTCC |
| ***rbp2b* F** | GACCTCAATCCCATAAAGTCCG |
| ***rbp2b* R** | CGTATGAATGTAAAGGATTCC |
| ***rbp2a* F (*Kpn*1)** | aagggtaccGAAATAAGGGAGAAAACCGACG |
| ***rbp2a* R (*Bam*H1)** | aagggatccCTAAGATGATTTTAGACCTTTCC |
| ***rbp2a* F (*Xho*1)** | aaggctcgagGAAATAAGGGAGAAAACCGACG |
| ***rbp2a* R (*Xba*1)** | aaggtctagaCTAAGATGATTTTAGACCTTTCC |
| ***rbp2b* F1 (*Kpn*1)** | aagggtaccGACCTCAATCCCATAAAGTCCG |
| ***rbp2b* R1 (*Bam*H1)** | aagggatccCGTATGAATGTAAAGGATTCC |
| ***rbp2b* F1 (*Xho*1)** | aaggctcgagGACCTCAATCCCATAAAGTCCG |
| ***rbp2b* R1 (*Xba*1)** | aaggtctagaCGTATGAATGTAAAGGATTCC |
